# Staurosporine-induced apoptosis and hydrogen peroxide-induced necrosis in two human breast cell lines

**DOI:** 10.1038/sj.bjc.6600675

**Published:** 2003-01-28

**Authors:** A L McKeague, D J Wilson, John Nelson

**Affiliations:** 1 School of Biology and Biochemistry, The Queen's University of Belfast, 97 Lisburn Road, Belfast BT9 7BL, Northern Ireland, UK; 2 School of Medicine/Anatomy, The Queen's University of Belfast, Northern Ireland, UK

**Keywords:** breast cancer, cell death assays

## Abstract

The use of apoptosis-inducing agents in the treatment of malignant cancer is increasingly being considered as a therapeutic approach. In this study, the induction of apoptosis and necrosis was examined in terms of temporal dose responses, comparing a malignant and nonmalignant breast cell line. Staurosporine (SSP)-induced apoptosis and H_2_O_2_-induced necrosis were evaluated by two cytotoxicity assays, neutral red (NR) and methyl-thiazolyl tertrazolium (MTT), in comparison with a differential dye uptake assay, using Hoechst33342/propidium iodide (Hoechst/PI). Confirmatory morphological assessment was also performed by routine resin histology and transmission electron microscopy. Cell viability was assessed over a 0.5–48 h time course. In nonmalignant HBL-100 cells, 50 nM SSP induced 100% apoptosis after a 48 h exposure, while the same exposure to SSP caused only 4% apoptosis in metastatic T47D cells. Although complete apoptosis of both cell lines was induced by 50 *μ*M SSP, this effect was delayed in T47D (24 h) compared with HBL-100 (4 h). Results also showed that neither MTT or NR can distinguish between the modes of cell death, nor detect the early onset of apoptosis revealed by Hoechst/PI.

In the normal breast, epithelial cell death by apoptosis occurs following lactation. This removal of secretory epithelial cells is necessary to remodel the breast to a quiescent organ of fat cells (Strange *et al*, 1992). In breast cancer, this delicate balance between a removal of cells through apoptosis and a replacement of cells via proliferation is disrupted (Martin and Green, 1995).

Identifying and counting apoptotic cells in culture can be difficult to do with accuracy, as each method available has advantages and disadvantages. A combination of these methods can give a more accurate picture of the apoptotic processes occurring in the cultured cell population.

For these reasons, we examined a combination of metabolic impairment, mitochondrial function and differential dye uptake assays, to compare the induction of apoptosis *vs* necrosis, by staurosporine (SSP) and H_2_O_2_, respectively. SSP, a potent protein kinase inhibitor with a broad spectrum of activity (Tamaoki *et al*, 1986; Kiyoto *et al*, 1987; Nakano *et al*, 1987), has been shown to induce apoptosis in a variety of human tumour cell lines (Bertrand *et al*, 1994). We used SSP to induce apoptosis in the nonmalignant human breast HBL-100 cell line (derived from a lactating breast and transformed by SV40 viral genome, Gaffney, 1984), and the T47D malignant breast cell line (derived from metastatic breast cancer, Keydar *et al*, 1979). The HBL-100 cell line is oestrogen receptor negative, and expresses relatively high levels of phospholipid/calcium-dependent protein kinase C (Bargou *et al*, 1995). The T47D cell line is oestrogen receptor positive and it also constitutively expresses protein kinase C isoforms (Nieves-Neira and Pommier, 1999). T47D Cells express transcripts for the apoptosis-promoting Bax gene but Bcl-2 transcripts are undetectable (Nieves-Neira and Pommier, 1999) or are present in low amounts; HBL-100 cells, on the other hand, display expression of both genes (Bargou *et al*, 1995). The T47D cells have point mutations in the core DNA-binding region of p53 (Nieves-Neira and Pommier, 1999), whereas HBL-100 cells exhibit wild-type p53 (Brodowicz *et al*, 2001).

The reactive oxygen species (ROS), H_2_O_2_, is produced during normal metabolism and also by phagocytic cells at the sites of inflammation (Hyslop *et al*, 1995). High levels of ROS can cause necrosis, while lower levels can cause apoptosis (Lennon *et al*, 1991; Dypbukt *et al*, 1994).

The neutral red (NR) metabolic impairment assay works simply on the principle that this dye accumulates in the lysosomes of viable cells by a combination of active endocytosis and pinocytosis until a stable equilibrium is reached. Dead cells lose their ability to accumulate and retain NR (Borenfreund and Puerner, 1985). However, this loss does not occur until late in the apoptotic process, when membrane integrity is compromised. Furthermore, this dye cannot distinguish between apoptotic or necrotic death (McCarthy and Evans, 1998).

The methyl-thiazolyl tertrazolium (MTT) assay measures the mitochondrial function activity of mitochondrial dehydrogenases (Twentyman and Luscombe, 1987). MTT is a yellow-coloured tetrazolium salt that is reduced to a purple formazan at the expense of the reduction reaction products with concomitant oxidation of NADH and NADPH (Altman, 1976). MTT does not distinguish between apoptosis and necrosis, nor does it take account of possible increases in cell number in a cycling cell population. So, in effect, cell populations may remain constant or even increase as the cell cycle progresses (McCarthy and Evans, 1998).

The DNA-binding dyes Hoechst 33342 and propidium iodide were first used together in a differential dye uptake assay (Hoechst/PI) on the fluorescent-activated cell sorter (FACS) (Sun *et al*, 1992). We have adapted the Hoechst/PI assay in microtitre plate format for microscopic identification and quantification of membrane integrity and nuclear morphology. Hoechst/PI works on the principle that Hoechst dye is rapidly incorporated into the nuclei of both apoptotic and necrotic cells where membrane integrity is compromised, while in live cells its incorporation into the nuclei occurs at a very slow rate. The nucleic acid dye PI, on the other hand, is excluded from both live and apoptotic cells and is incorporated into necrotic and secondary necrotic cells where membrane integrity is lost. PI causes the nuclei to stain bright orange/red (Nakajima *et al*, 1996). This loss of membrane integrity occurs very late in the apoptotic process but early during necrosis (Darzynkiewicz and Li, 1996). In viable cells, phosphatidyl serine (PS) is predominantly found on the inner leaflet of the plasma membrane. In apoptotic cells, PS is translocated to the outer leaflet and is displayed extracellularly. This forms the basis for detection of apoptosis using the PS-binding protein, annexin V. However, recent work shows that PS is also translocated in early necrosis (oncosis) (Lecoeur *et al*, 2001). Thus, as an indicator of apoptosis, annexin V labelling index must be interpreted with caution. In the light of this, we have examined differential dye uptake as an alternative, microtitre plate-based assay of apoptosis in adherent cell lines.

## MATERIALS AND METHODS

### Cells and experimental treatments

The HBL-100 cell line derived from the lactating breast (Gaffney, 1984) and the T47D breast carcinoma cell line (Keydar *et al*, 1979) were grown in Dulbecco's modified Eagle's medium (DMEM) with 0.11 g l^−1^ sodium pyruvate and pyroxidine (Life Technologies) supplemented with 10% fetal calf serum (PAA Laboratories) and 1% penicillin/streptomycin (Life Technologies). Cells were cultured in a humidified 5% CO_2_ atmosphere at 37°C.

Cells were seeded at a density of 2×10^4^ cells well^−1^ and for approximately 16 h before treatments were added. Experimental treatments of 50 *μ*M SSP, 50 nM SSP and 3 mM H_2_O_2_ in growth medium were added to both cell lines for a time course ranging from 0.5 to 48 h. A control of growth medium was also run in parallel for each time period in both cell lines. After treatments, the cells were processed in accordance with each particular assay.

For confirmatory morphological and ultrastructural studies, semiconfluent cultures of both cell lines were exposed to treatments of 50 *μ*M SSP or 3 mM H_2_O_2_ in growth media for 24 h. After treatment, cells were rinsed in calcium-free saline, harvested mechanically and then pelleted in 1.5 ml Eppendorf tubes. The cell pellets were fixed overnight in 3% glutaraldehyde in 0.1 M cacodylate buffer. They were then washed several times in 0.1 M cacodylate buffer, postfixed in 0.1 M osmium tetroxide in 0.1 M cacodylate buffer and dehydrated in a graded series of ethanol before embedding in epoxy resin. Semithin sections (1 *μ*m) of the cell pellets were cut using a Reichert–Jung Ultracut-E microtome. The sections were stained with toluidine blue and examined using a Leitz Diaplan, microscope, and representative sections photographed. Ultrathin sections (70–90 nm) were cut and stained with uranyl acetate and lead citrate (Reynolds, 1963), examined using a JEOL CX 100 electron microscope and representative fields photographed.

### NR assay

This assay was performed according to the method of Borenfreund and Puerner (1985) with modifications as detailed: a 0.4%. NR stock solution was diluted 1 : 80 in growth medium to a final concentration of 50 *μ*g ml^−1^. Control medium was removed from the 96-well microtitre plates and replaced with 200 *μ*l well^−1^ of NR solution and incubated for 4 h at 37°C. The NR solution was aspirated and wells were rinsed once with 200 *μ*l well^−1^ of 4% formaldehyde containing 1% calcium chloride. Finally, 200 *μ*l well^−1^ of solubilisation fluid (1 ml of glacial acetic acid in 100 ml of 50% ethanol) was added and agitated for 15 min on a microtitre plate shaker. The absorbance was read at 550 nm and for each treatment six replicate wells were examined.

### MTT assay

This assay was performed according to the modified method of Twentyman and Luscombe (1987) as detailed: 10 *μ*l well^−1^ of a 5 mg ml^−1^ stock MTT solution was added to cells in a 96-well microtitre plate previously seeded at a volume of 100 *μ*l well^−1^. This was incubated for 2 h at 37°C until the purple formazan crystal developed. Finally the MTT-containing medium was removed and 200 *μ*l of dimethyl sulphoxide (DMSO) was added to each well. The absorbance was read at 550 nm and for each treatment six replicate wells were examined.

### Hoechst 33342/PI assay

This assay was performed according to a combination of methods (Dive *et al*, 1992; Ormerod *et al*, 1992; Skehan *et al*, 1993) with modifications as detailed: 50 *μ*l well^−1^ of a 200 *μ*g ml^−1^ PI stock (a final concentration of 40 *μ*g ml^−1^) and 2.5 *μ*l of a 100 *μ*g ml^−1^ Hoechst 33342 was added to a microtitre plate well volume of 100 *μ*l, and incubated in the dark for 60 and 15 min respectively. A 100 *μ*l of methanol : acetic acid (3 : 1) fixative was then added directly to each well. Cells were viewed under a UV microscope with DAPI filter. Six replicate wells were analysed for each treatment by quantitative and qualitative examination of four random fields in each well.

## Results

**Figure 1:**
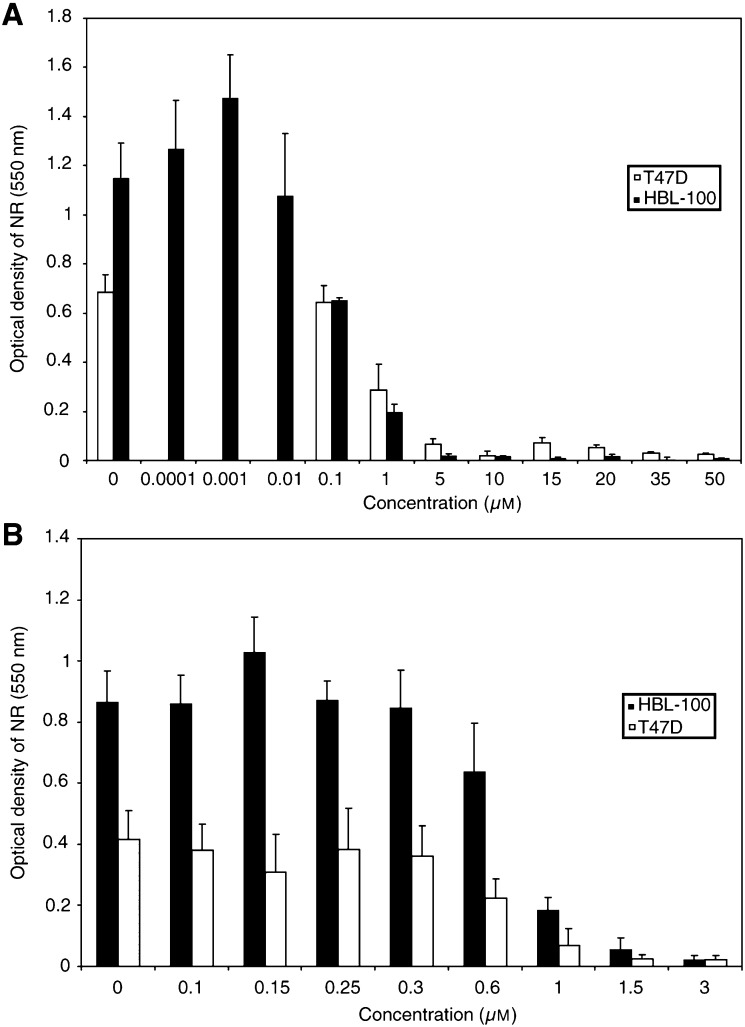
Dose response assays (assessed by NR) for the HBL-100 and the T47D cell lines over a 24 h period. (**A**) Dose response of SSP-treated cells using the range from 10 pM to 50 *μ*M. LD_100_ (no significant fraction of viable cells remaining)=50 *μ*M. (**B**) Dose response of H_2_O_2_-treated cells using the range from 0.1 to 3 mM. The LD_100_ (no significant fraction of viable cells remaining)=3 mM.

**Figure 2:**
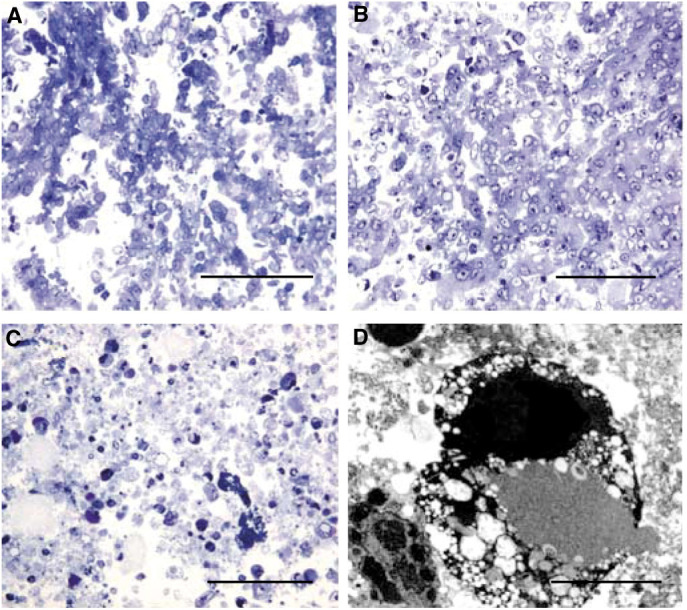
Semithin resin sections (1 *μ*m) stained with toluidine blue to show morphology of control HBL-100 cells (**A**), after 24 h treatment with 3 mM H_2_O_2_ (**B**), or 50 *μ*M SSP (**C**). The H_2_O_2_-treated cells appeared swollen, the cell membranes disrupted and the nuclei appeared pyknotic, whereas the SSP-treated cells were shrunken with condensed nuclear material. At the ultrastructural level, the nuclear material appeared to have fragmented to give electron dense bodies (**D**). Magnification bars **A**–**C**=0.1 mm, **D**=5  *μ*M.

Pilot dose responses were performed using the NR assay to assess an approximate lethal dose 100% (LD_100_) of SSP and H_2_O_2_ in both cell lines. From the results, it was concluded that in both cell lines the approximate LD_100_ doses for SSP and H_2_O_2_ were 50 *μ*M and 3 mM, respectively (Figure 1

). SSP at 50 nM was chosen for comparison with the 1000-fold higher SSP dose. The morphological examination of the cells after these doses confirmed the NR findings. For example, HBL-100 cells treated with H_2_O_2_ at 3 mM were swollen with evidence of cell membrane disruption. The nuclei appeared pyknotic and there was considerable cellular debris in the pellets when compared to the control sections (Figure 2A, B

). Treatment with SSP at 50 *μ*M resulted in cells exhibiting a different overall morphology: they were shrunken with condensed nuclear material (Figure 2C), which had fragmented into electron dense bodies (Figure 2D).

**Figure 3:**
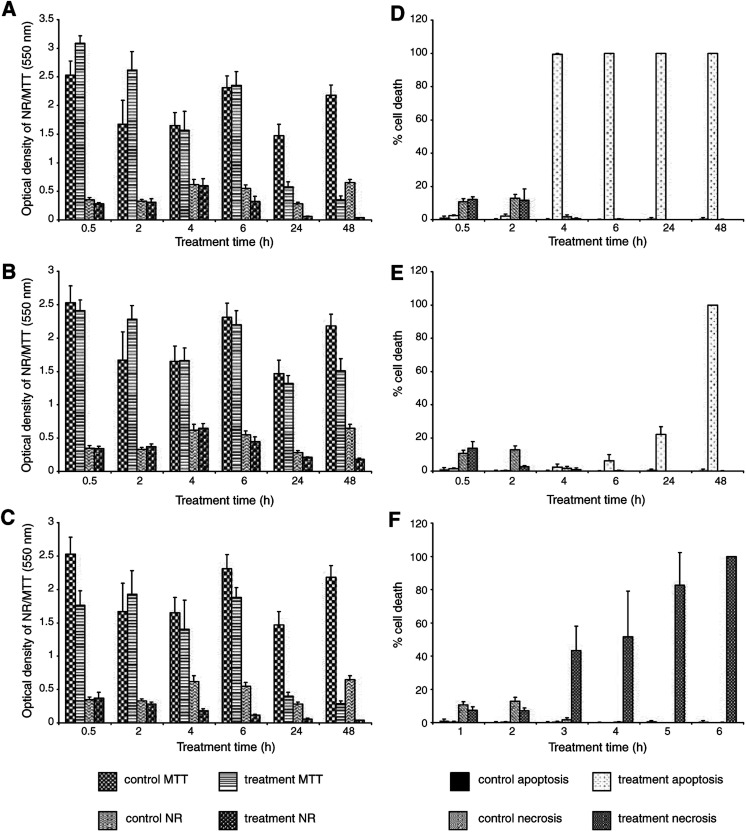
Comparative analysis of the cytotoxicity assays (MTT and NR) *vs* the differential dye uptake assay (Hoechst/PI) in the HBL-100 cell line. Graphs (**A**) 50 *μ*M SSP, (**B**) 50 nM SSP and (**C**) 3 mM H_2_O_2_ represent the results of the MTT and NR assays over the 0.5–48 h time course. Graphs (**D**) 50 *μ*M SSP, (**E**) 50 nM SSP and (**F**) 3 mM H_2_O_2_ represent the results of the percentage of apoptotic and necrotic cell death detected by the Hoechst/PI assay over the 0.5–48 h time course. The values of 100% cell death represent when the entire population of cells analysed were apoptotic (graphs **D** and **E**) or necrotic (graph **F**).

A comparison was made between all assays and treatments used for the HBL-100 cell line (Figure 3

). The results for the MTT and NR assays for the 50 *μ*M SSP treatment showed that it was 24 h before that there was a marked increase in cell death compared with control levels (Figure 3A). The extent of apoptosis *vs* necrosis assessed by the Hoechst/PI assay was compared using the same dose of SSP over the 0.5–48 h time course (Figure 3D). Apoptosis did not gradually increase in the first 4 h. Instead, at this time point, virtually the whole population became apoptotic.

The MTT and NR results for the 50 nM SSP treatment revealed that a marked increase in cell death did not occur until 48 h and the percentage of cell death was not as high as that observed with the 1000-fold higher dose of SSP (Figure 3B). The Hoechst/PI results showed a marked progression of apoptosis from 6 to 48 h (Figure 3E). With 50 nM SSP the onset of apoptosis appeared to be a slower process compared with the 1000-fold higher dose, and the percentage of dead cells was correspondingly lower at the 24 h time period.

MTT and NR results for the 3 mM H_2_O_2_ treatment reveal that a marked increase in cell death was observed in the NR assay as early as 4 h, yet it was 24 h before this was observed with MTT (Figure 3C). Hoechst/PI assay results showed a steady progression in necrosis over the entire exposure time (Figure 3F), and virtually no apoptosis was observed.

**Figure 4:**
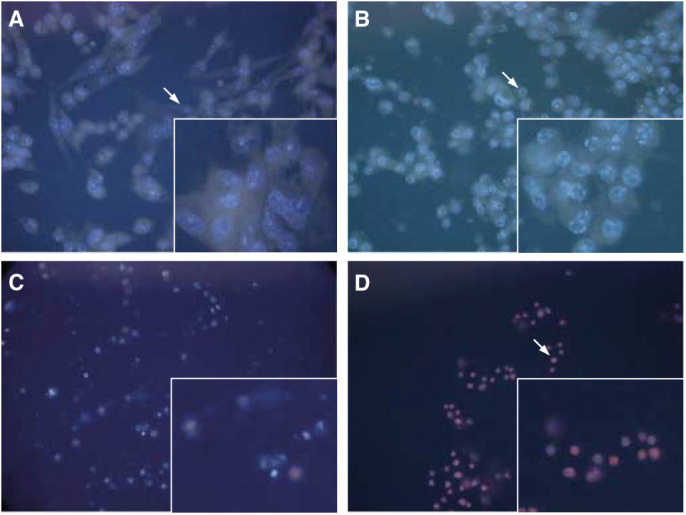
Hoechst/PI staining for the HBL-100 cell line showing (**A**) typical untreated cells after 6 h (arrow). Inset shows higher magnification of control cells displaying nuclei with normal morphology. (**B**) SSP-treated cells (50 *μ*M) after 4 h displaying chromatin margination (arrow, and detail of cells in inset) characteristic of apoptosis. (**C**) SSP-treated cells (50 nM) after 48 h showing typical end-stage apoptosis. Inset shows higher magnification of secondary necrotic cells. (**D**) H_2_O_2_-treated cells (3 mM) after 24 h showing cells infiltrated by PI (arrow, and details of cells in inset).

A population of untreated control cells at the 6 h time course period was chosen for comparison (Figure 4A

) with Hoechst/PI differential dye uptake images for each treatment used in the HBL-100 cell line. This time point was found to be representative of the control cell population at all the time points examined and was chosen because the time to maximum effect varied in the treatment groups. The extent of apoptosis after 4 h of the 50 *μ*M SSP dose was evinced by typical chromatin margination in the cell nuclei of virtually all cells, as well as a reduction in cell volume (Figure 4B). After 48 h of exposure to 50 nM SSP, only a small proportion of the cell population survived (Figure 4C). The remaining cells showed few features of apoptosis, instead cells appear to have undergone secondary necrosis. At the 24 h time period, 3 mM H_2_O_2_-treated cells were entirely infiltrated by PI, consistent with necrosis.

**Figure 5:**
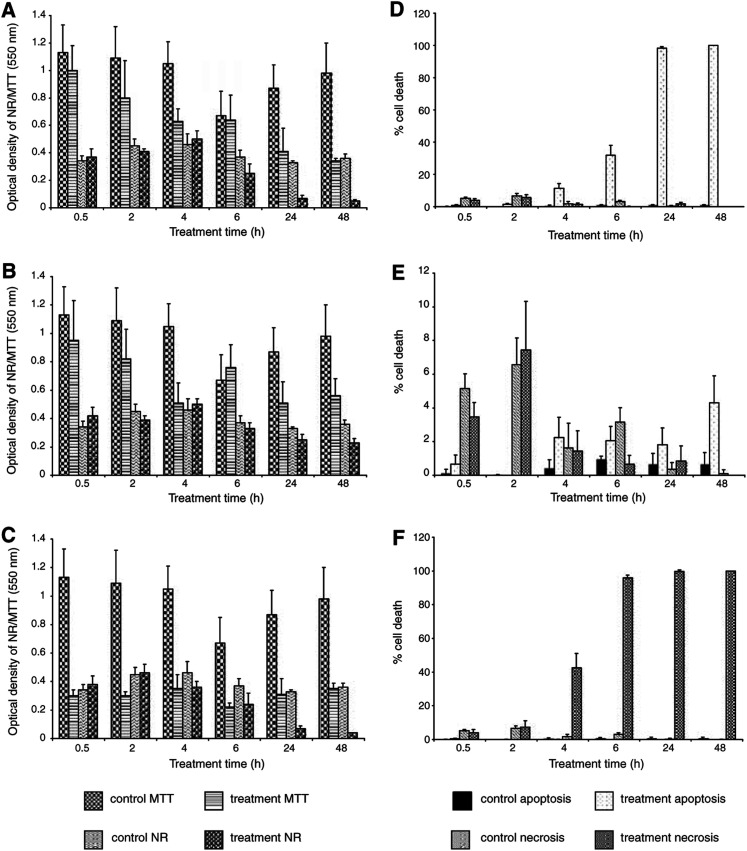
Comparative analysis of the cytotoxicity assays (MTT and NR) *vs* the differential dye uptake assay (Hoechst/PI) in the T47D cell line. Graphs (**A**) 50 *μ*M SSP, (**B**) 50 nM SSP and (**C**) 3 mM H_2_O_2_ represent the results of the MTT and NR assays over the 0.5 to 48 h time course. Graphs (**D**) 50 *μ*M SSP, (**E**) 50 nM SSP (note the scale on the ordinate axis in this figure) and (**F**) 3 mM H_2_O_2_ represent the results of the percentage of apoptotic and necrotic cell death detected by the Hoechst/PI assay over the 0.5–48 h time course. The values of 100% cell death represent when the entire population of cells analysed were apoptotic (graph **D**) or necrotic (graph **F**).

Comparison between all assays and treatments using the T47D cell line showed that the 50 *μ*M SSP treatment resulted in a marked increase in cell death (Figure 5A

): this was not observed in either MTT or NR assays until 24 h. Hoechst/PI assay for the same SSP dose showed a graduated increase in apoptosis up to 24 h (Figure 5D), at which point maximum apoptosis was observed.

With the 50 nM SSP treatment, it was 24 h before MTT cell death was observed and 48 h before this was seen in NR (Figure 5B). The Hoechst/PI results for this dose of SSP showed that from 4 h onwards, the cells maintained a uniform percentage of apoptotic cells, which began to increase further at 48 h (Figure 5E). From 0.5 to 4 h, there was low level of necrosis in the SSP-treated cells, but this was not significantly higher than the control cells. There was a steady level of necrosis until the number of apoptotic cells within the population increased at 48 h.

A marked and steady increase in cell death was seen with Hoechst/PI throughout the entire time course study using 3 mM H_2_O_2_. However, it was not until 24 h that cell death was observed by NR (Figure 5C). Apoptosis (as judged by Hoechst/PI) was virtually absent. However, necrosis appears at a marked level by 4 h and peaked by 6 h (Figure 5F).

**Figure 6:**
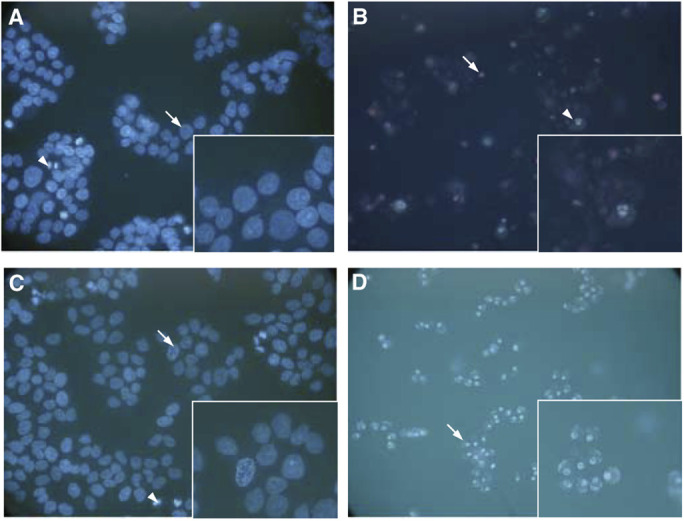
Hoechst/PI staining for the T47D cell line showing (**A**) typical untreated cells after 6 h (arrow and higher power details in inset) and cells undergoing mitosis (arrowhead). (**B**) SSP-treated cells (50 *μ*M) after 24 h showing cells undergoing secondary necrosis (arrow) and those in typical apoptosis (arrowhead and detailed in inset). (**C**) SSP-treated cells (50 nM) after 24 h displaying an apoptotic cell (arrow and inset showing higher power of apoptotic cell) and mitotic cell (arrowhead). (**D**) H_2_O_2_-treated cells (3 mM) after 24 h showing the characteristic halo pattern (arrow) displayed by ‘ghost’ cells (seen at higher magnification in inset).

Images of Hoechst/PI differential dye uptake for each treatment used in the T47D cell line were compared to untreated cell population. The 6 h time point was typical (Figure 6A

) of the control populations examined throughout the time course, in which cells undergoing different phases of mitosis were observed. Treatment of cells with 50 *μ*M SSP for 24 h (Figure 6B) resulted in a marked increase in secondary necrosis in the apoptotic population. After 24 h, 50 nM SSP-treated cells showed a small percentage of cell death (Figure 6C), but no significant change was observed in the overall cell population. Some mitotic figures could still be seen. H_2_O_2_ (3 mM) treatment after 24 h showed an entire cell population with no distinct nuclear staining (Figure 6D): such ‘ghost’ profiles have been reported to contain single or multiple clumps of rounded, bright and condensed chromatin within a pale cytoplasm (Edwards and Tolkovsky, 1994).

## Discussion

This study has evaluated the use of cytotoxicity assays in comparison with a differential dye uptake assay. Previous studies have used one or both of these cytotoxicity assays and/or the differential dye uptake assay in combination with other assay methods (Edwards and Tolkovsky, 1994; Hardwick *et al*, 1996; Hughes *et al*, 1996; Burger *et al*, 1999; Lizard *et al*, 1999; McGuinness *et al*, 1999; Sumitomo *et al*, 1999; DeMeester *et al*, 1997). However, none have used this specific combination of assays in evaluating apoptosis in breast cancer cell lines. In addition, the present study has allowed comparison of the induction of apoptosis between a nonmalignant and metastatic breast cell line.

We showed that the time of onset of apoptosis as judged by Hoechst/PI does not correlate with any marked cell death detected with either cytotoxicity assay. At 50 *μ*M SSP, the nonmalignant HBL-100 cell line exhibits approximately 100% apoptosis at 4 h (as judged by Hoechst/PI). However, the MTT and NR assays do not detect cell death until 24 h. In contrast to the HBL-100 line, the metastatic T47D cells show only 15% apoptosis at 4 h. Although they become 100% apoptotic at 24 h, MTT and NR show only 50% cell death at that time point. At this 50 *μ*M SSP dose, it was observed in both cell lines that a reduction in MTT correlated with end-stage apoptosis (secondary necrosis) as detected by Hoechst/PI. The onset of apoptosis as measured by Hoechst/PI was not reflected by NR either. This was perhaps not surprising, as NR indicates when membrane integrity is lost, late in the apoptotic process.

When the HBL-100 cell line was treated with 50 nM SSP, it yielded similar results to that for 50 *μ*M SSP, again marked changes in MTT and NR reflected end-stage apoptosis (as judged by Hoechst/PI). The T47D cell line yielded different results for the 50 nM SSP dose, as an initial reduction in MTT at 4 h correlated with the observation of apoptosis as detected by Hoechst/PI coincidentally. However, this appeared to vary over the time course and no marked increase in initial apoptosis levels, detected by Hoechst/PI), was seen even after 48 h. This can perhaps be explained by the greater number of mitotic cells observed (data not shown) and is therefore reflective of the cycling cell population. This result also highlights the low level of apoptosis induced by the 50 nM SSP dose in the metastatic breast cell line (approximately 4%) as compared with the non-malignant cell line (approximately 100%), even after 48 h of treatment.

The necrotic treatment, 3 mM H_2_O_2_, in the HBL-100 cell line showed that by 4 h there was a decrease in the amount of viable cells as detected by NR; however, it was 24 h before this was observed by MTT. Interestingly, the percentage of necrosis detected by Hoechst/PI increased steadily from as early as 4 h.

The T47D cell line gave a very different result with this treatment, as MTT showed a steady decrease in the number of viable cells detected (compared with control levels) from as early as 0.5 h. It was 24 h before this type of decrease was detected by NR. The MTT result may be explained by the possibility that the metastatic cell line contains a subpopulation of cells that are more resistant to necrotic cell death.

In conclusion, the 50 nM SSP dose showed that the metastatic cell line is 25 times more resistant to apoptosis than the nonmalignant cell line. The T47D cells when treated with 50 *μ*M SSP, revealed a delay in reaching approximately 100% apoptosis (by 24 h) as compared with the HBL-100 cells (by 4 h). Results of the MTT assay in combination with the appearance of secondary necrosis (as judged by Hoechst/PI) suggest that complete mitochondrial breakdown (as opposed to membrane pore transition) occurs only when secondary necrosis has begun. The 3 mM H_2_O_2_ results also suggest that the T47D cell line is more resistant to necrotic cell death than the HBL-100 cell line. Comparing these assay methods has thus allowed for a quantitative assessment of apoptosis in these breast cell lines. In addition, this combination of assay methods, with particular emphasis on Hoechst/PI, has also shown its ability to distinguish between apoptosis and necrosis *in vitro*.

In terms of relevance to cancer biology, the results have significance: they suggest that malignant breast cells are more resistant than nonmalignant cells to apoptotic induction, although possible differences in bcl-2 gene expression or other targets of SSP would also have an influence. Potential therapeutic approaches using drug-induced cell death (Makin and Dive, 2001) would need to be of sufficient duration and dose to overcome this resistance. However, more breast cell lines of both normal and malignant phenotype would need to be evaluated to confirm the assertion that malignant breast cells are more resistant to apoptotic induction.
